# Editorial: Cell death in cancer immunology

**DOI:** 10.3389/fonc.2024.1504738

**Published:** 2024-11-19

**Authors:** Laura Mondragón, Shensi Shen, Laura Senovilla

**Affiliations:** ^1^ T Cell Lymphoma Group, Josep Carreras Leukaemia Research Institute (IJC), Badalona, Spain; ^2^ Institute of Thoracic Oncology and Department of Thoracic Surgery, National Clinical Research Centre for Geriatrics, West China Hospital, Sichuan University, Chengdu, China; ^3^ Unidad de Excelencia Instituto de Biomedicina y Genética Molecular (IBGM), Universidad de Valladolid-Spanish National Research Council (CSIC), Valladolid, Spain; ^4^ Centre de Recherche des Cordeliers, Equipe Labellisée par la Ligue Contre le Cancer, Université Paris Cité, Sorbonne Université, Inserm U1138, Institut Universitaire de France, Paris, France; ^5^ Metabolomics and Cell Biology Platforms, Institut Gustave Roussy, Villejuif, France

**Keywords:** immunotherapy, immunogenic cell death, prognosis predictive models, biomarkers, vaccines

The relationship between cell death and cancer immunology has been studied for decades with different approaches, as cell death plays a crucial immunomodulatory role in tumor initiation, progression and metastasis (1). Research has focused on understanding the immune system’s role in tumor cell resistance to cell death; discerning how cell death paradoxically promotes immunosuppression and tumor progression; explaining the mechanisms by which cell death induces tumor immunogenicity; studying the immunomodulatory effects of dead or dying tumor cells on tumor microenvironment; and investigating how various immunotherapies trigger tumor cell death.

Given the complex interplay between cancer immunology and cell death, this Research Topic of fourteen articles highlights the latest advances in the field through three reviews and eleven original studies. These works can be categorized into two main axes: 1) the development of prognostic models using machine learning, multiomics and patient datasets, and 2) the identification and validation of new biomarkers and therapies focused on the activation of the immune system.

In the last decades, the rise of “omic” technologies has enabled the development of clinical prediction models aimed at improving medical decision-making, enhancing patient’s outcomes, and identifying novel biomarkers and therapeutic targets. In the on-going quest for better treatments and ways to overcome cancer cell death resistance and immune evasion, new cell death mechanisms linking these processes have been discovered, offering fresh new strategies to combat cancer.

This is the case of disulfidptosis, a recently described new form of cell death caused by disulfide stress (2). Disulfides are produced in response to oxidative stress to help maintain the secondary, tertiary, and quaternary structures of proteins by acting as inter- and intra-subunit cross-links. Excessive intracellular accumulation of disulfides solute carrier family 7 member 11 (SLC7A11) induces, together with glucose starvation, aberrant disulfide bonds formation between actin cytoskeleton, causing its collapse in a particular and orderly manner. Overexpression of SLC7A11 and GLUT inhibitors has proved to inhibit tumor growth, which may contribute to the development of a new therapeutic strategy against cancer.

Consequently, multiple works have been undertaken to answer these questions in the last months, mostly by predictive models development. In this sense, Li et al. make use of 20 diagnosed-related groups (DRGs) and LASSO and Cox regression analysis to provide, among other immune cell infiltration patterns, a new potential therapeutic target: POU Class 4 Homeobox 1 (POU4F1), which promotes cell proliferation, migration and, most importantly, disulfidptosis in colon adenocarcinoma (COAD) patients. In a broader application, Wang et al. also develop a similar analysis making use of disulfidptosis-related genes identified from CRISPR–Cas9 screenings leading to the identification of neuronal acetylcholine receptor subunit alpha-5 (CHRNA5) as a potential therapeutic target due to its impact on cell proliferation, migration, and disulfidptosis in the context of lung adenocarcinoma (LUAD). Finally, Zhang et al. focused on Methods Public datasets to develop a prognostic model for LUAD to predict patient’s survival and the efficacy of immune checkpoint blockage considering the expression of disulfidptosis-related genes.

Yang et al. also explore disulfidptosis and immune microenvironment to develop a new prognostic model to identify therapeutic targets for hepatocellular carcinoma (HCC) utilizing bulk ribonucleic acid (RNA) sequencing, spatial transcriptomic (ST) and single-cell RNA sequencing. Their findings reveal that N-myc downregulated gene 1 (NDRG1) influences macrophage differentiation and enables tumor cells to evade the immune system. Similarly, Zheng et al. leverage RNA sequencing data and clinical information from HCC patients in the *The Cancer Genome Atlas* (TCGA) to create a predictive model for chemotherapy sensitivity and immunotherapy efficacy in HCC.

To finish the first axis, ferroptosis, a form of cell death dependent on iron and characterized by the accumulation of lipid peroxides, and fatty acid metabolism (FAM) in the tumor microenvironment (TME), is reviewed by Guo et al. in the context of ovarian cancer and its role in tumor suppression (3, 4). Ferroptosis role in the onset, progression, and incidence of ovarian cancer and their synergy with immunotherapy is defined together with new potential treatments based on these facts. Then, Zhu et al. explore the relationship between ferroptosis and patients outcome in colorectal cancer (CRC) with the objective of anticipating immunotherapy effectiveness. They make use of TCGA and GEO databases to create the FeFAMscore, which proved that ferroptosis regulators and FAM-related genes not only enhance immune activation, but they also contribute to immune escape. Finally, Wang et al. describe the generation of the PANoptosis-model, based on PANoptosis cell death, which is initiated by innate immune sensors and driven by caspases and receptor-interacting protein kinases (RIPKs) through the so-called PANoptosome complexes. They make use of clinical and single-cell data from breast cancer patients and validate them by means of immunohistochemistry (IHC) assays, proving how this model can help in predicting breast cancer prognosis and treatment personalization.

Regarding the second set of articles of this Research Topic, Shabrish et al. takes profit of cell-free chromatin particles (cfChPs) circulating in blood of cancer patients or that have been released by dying cancer cells, which upon internalization by healthy cells can modulate the activation of immune checkpoints, providing a novel form of immunotherapy of cancer. Verhaar et al. make use of MHC class I chain related-proteins A and B (MICA, MICB) glycoproteins present on the surface of epithelial and hematopoietic cancer cells and that bind to natural killer group 2D (NKG2D) and activate the immune system. By means of nanobodies, Verhaar et al. surface-dispose MICA together with the Maytansine derivative DM1, selectively killing MICA positive tumor cells *in vitro*.

Additionally, with the goal of providing a long-term cancer protection, Wang et al. review therapeutic strategies to enhance the immunogenicity of dying tumor cells leading to achieve more effective and sustained immune activation. They focus on strategies for inducing ICD and designing vaccines that introduce more immunogenic antigens and stimulating factors. Complementing this review, Budhu et al. provide a practical approach, addressing the common issue of immunotherapy failure due to the lack of immune infiltrates in tumor. They compare the effectiveness of anti-PD1 therapy combined with radiation therapy (RT), vascular targeted photodynamic therapy (VTP) and cryoablation (Cryo), demonstrating that these tumor destruction methods can indeed improve therapy outcomes by eliciting different immune responses.

Taking into account the effect of damage-associated molecular patterns (DAMPs) and certain cytokines and chemokines in ICD, Naessens et al. explore the intricate role of CX3CL1 in immunogenic apoptosis induced by mitoxantrone (MTX) in cancer cells. CX3CL1, which exerts a role in cellular signaling and immune cell interactions, has been denoted as a “find me” signal that stimulates chemotaxis of immune cells towards dying cells, facilitating efferocytosis and antigen presentation. Its role in ICD in melanoma and fibrosarcoma cells is described by studying its role upon the activation of an adaptive immune response against cancer cells undergoing ICD.

To conclude this Research Topic focused on non-invasive biomarkers for better diagnostics and prognostics in CRC and liver cancer, de Castro et al. provide a revision on advanced cytometry panels covering over 40 parameters (computational cytometry) that make use of elemental mass spectrometry to detect metal-conjugated antibodies bound to antigens of interest on single cells. This next generation flow cytometry platform can be applied to the study of the immune system and the search of novel biomarkers to aid in diagnosis and prognosis, and to even predict clinical response to different treatments.

We believe that, collectively, these fifteen contributions in “Cell Death in Cancer Immunology” will provide a comprehensive description of current genomic strategies for the development of new prognostic methods based on the study of molecular, immunological, and therapeutic aspects of cancer cell death, and potential development of new biomarkers for therapeutic gain in the context of immune system activation.
